# Process Design of Aluminum Tailor Heat Treated Blanks

**DOI:** 10.3390/ma8125476

**Published:** 2015-12-09

**Authors:** Alexander Kahrimanidis, Michael Lechner, Julia Degner, Daniel Wortberg, Marion Merklein

**Affiliations:** 1 Daimler AG, Process Development and Materials, HPC 050-F155, 71059 Sindelfingen, Germany; alexander.kahrimanidis@daimler.com (A.K.); daniel.wortberg@daimler.com (D.W.); 2 Institute of Manufacturing Technology, Friedrich-Alexander-Universität Erlangen-Nürnberg, 91058 Erlangen, Germany; michael.lechner@fau.de (M.L.); julia.degner@fau.de (J.D.)

**Keywords:** lightweight design, tailor heat treated blanks, material model

## Abstract

In many industrials field, especially in the automotive sector, there is a trend toward lightweight constructions in order to reduce the weight and thereby the CO_2_ and NO*_x_* emissions of the products. An auspicious approach within this context is the substitution of conventional deep drawing steel by precipitation hardenable aluminum alloys. However, based on the low formability, the application for complex stamping parts is challenging. Therefore, at the Institute of Manufacturing Technology, an innovative technology to enhance the forming limit of these lightweight materials was invented. The key idea of the so-called Tailor Heat Treated Blanks (THTB) is optimization of the mechanical properties by local heat treatment before the forming operation. An accurate description of material properties is crucial to predict the forming behavior of tailor heat treated blanks by simulation. Therefore, within in this research project, a holistic approach for the design of the THTB process in dependency of the main influencing parameters is presented and discussed in detail. The capability of the approach for the process development of complex forming operations is demonstrated by a comparison of local blank thickness of a tailgate with the corresponding results from simulation.

## 1. Introduction

**Figure 1:**
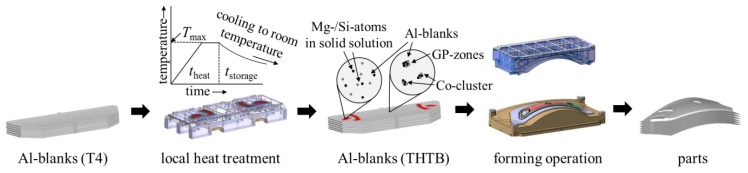
Principal integration of Tailor Heat Treated Blanks (THTB) technology in the process chain.

Lightweight design is a promising approach to reduce the overall emissions caused by transportation [1]. In recent years, aluminum alloys have drawn a lot interest due to their high strength-to-density ratio. For outer panel applications in passenger cars, mainly AlMgSi-alloys (6xxx-series) are used [2] because of their high surface quality. However, the forming behavior of these alloys must be considered as limited, especially when compared to conventionally used, mild or high strength steels [3]. An auspicious strategy to overcome these forming limitations is the use of so called tailor heat treated blanks (THTB). The concept is based on introducing a strength and ductility distribution to the semi-finished blank that is optimized with regard to a subsequent forming step [4]. Hereby, the local adjustment of the mechanical properties is achieved by a short-term heat treatment before the stamping operation. [5]. The principle integration in the press shop is presented in Figure 1. The blanks are delivered in state T4 and exposed to the heat treatment uncoupled from the press line. The THTB with the defined and graded mechanical properties are then stored at room temperature until they get processed in a forming operation. 

The resulting time–temperature profile can be characterized by the heating time, the holding time, the cooling time and the maximum temperature. Former investigations have identified that the maximum temperature is the most important parameter for the mechanical properties [6]. Concerning the technical application and the necessary localization of the heat treatment, the heating and holding time should be as short as possible. Based on the high heat conductivity the self-quenching effects can be used and no active cooling is mandatory. For 6xxx-series aluminum alloys, decreasing yield strength with increasing maximum temperature is observed. On a microstructural basis, this softening is explained by the dissolution of the strength increasing co-clusters and GP-zones [7]. In this quasi-solution annealed state, the dislocations are no longer hindered in their movement and an earlier plastification of the material can be identified. Immediately after the heat treatment, a natural ageing process is observed. As a consequence, the local material behavior during forming is mainly defined by a combination of heating parameters and ageing time. In order to enhance the formability, the heating zones must be chosen with the intent to improve the material flow to crack critical areas. For complex forming operations, this definition is described as the most challenging step of the THTB technology [8]. In the literature, inverse strategies with iterative optimization algorithms have shown the most promising results [9]. For a reliable numerical simulation it is necessary to describe the elastic-plastic as well as the failure behavior in dependency of the heat treatment and the storage of the blanks. However, so far no holistic approach is available that provides a unified description. Therefore, within this article, a new process design approach will be introduced in order to describe the yield surfaces, flow curves and forming limit diagrams for THTB. Thus, theoretical models will be used for the application of the THTB technology to a complex forming part. 

## 2. A Material Model for Tailor Heat Treated Blanks

In the following section, the approach is first introduced in a generalized formulation that allows using any common yield function and hardening law. Limitations are discussed alongside with the possibility to enhance the model by considering additional process variables. In a second step, an experimental dataset of a fast hardenable (PX) AA6014 alloy is analyzed and the THTB material model is applied. The maximum temperature and ageing time are implemented as process variables. Finally, the application of the fundamental assumptions, the material model and the implementation in to the simulation will be validated by heat treatment and stamping of a tailgate. In addition, the theory of Marciniak-Kuczyński (MK model) [10] is applied to calculate a forming limit curve that is used for post-forming analysis of the part.

### 2.1. General Approach to Describe the Plastic Behavior of Tailor Heat Treated Blanks (THTB)

**Figure 2:**
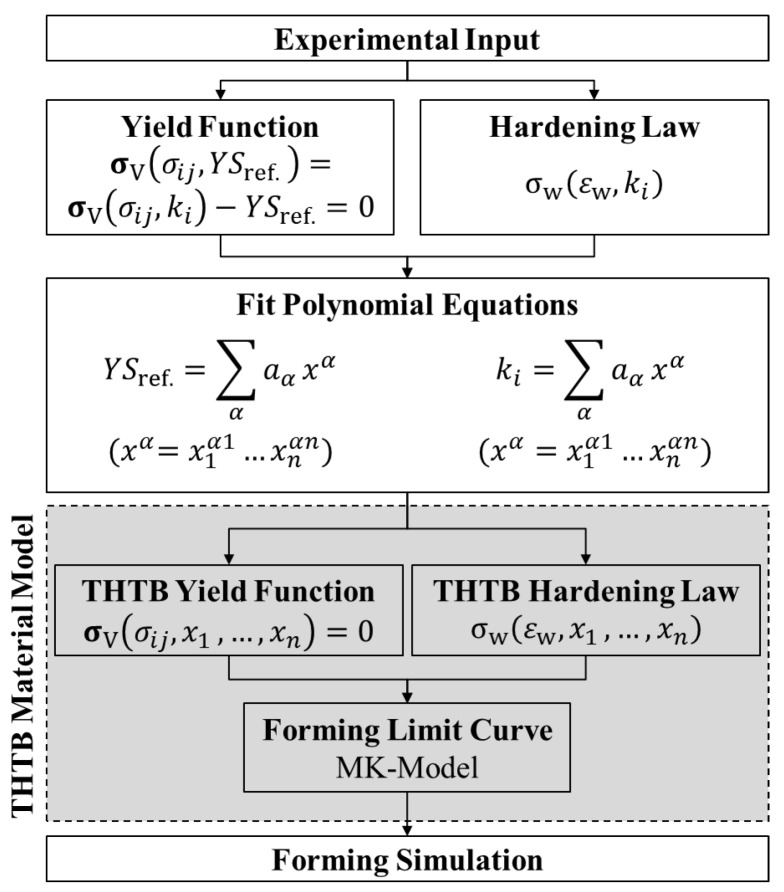
General approach to describe the plastic behavior of THTB.

The plastic behavior of a material can be described with a yield surface σ_V_ and a flow curve σ*_w_* [11]. The yield surface provides the onset of plastic deformation depending on the stress state and a yield parameter *YS_ref_.* Thereby, the stress state is defined by the normalized components of the stress tensor σ*_i_*_= 1,2 *j* = 1,2_, while the yield parameter is an experimental value, e.g., the uniaxial yield strength under load parallel to the rolling direction. The yield surface is typically modeled by a function based on experimental input. The number of experiments necessary for the calibration varies depending on the chosen yield criterion, e.g., 3 for Hill48 [12] or 8 for BBC2005 (Banabic-Balan-Comsa) [13]. The flow curve describes the hardening of the material as soon as plastic deformation is reached and is commonly described with a hardening law, which is a function of true plastic strain ε*_w_*. Over the last decades, various phenomenological yield functions and hardening laws have been published that can be used to define the mechanical behavior of materials [14]. What all of these models have in common is that they require experimental input and are dependent on functional parameters *k_i_* that are often determined by an iterative algorithm, e.g., minimizing the sum of squared residuals. Vogt used this in the context of THTB and showed for the Hockett-Sherby hardening law [15] that all its functional parameters can be expressed based on the maximum temperature [16]. During the investigations, the parameters of the Hockett-Sherby law were replaced with polynomial equations that represented the change with maximum temperature. However, this simple model is not able to describe the onset of plastic deformation and the failure behavior of the material in dependency of the heat treatment. Moreover, the storage of the blanks and thereby the ageing of the blanks is not taken into account. An overview of the suggested approach to model the material behavior of THTB is given in Figure 2.

The experimental input represents the nodes of the THTB model and is thus determined for different combinations of the process variables *x_n_* which influence the mechanical properties e.g., maximum temperature, ageing time, heating time, cooling conditions, *etc.* In the next step, a particular solution is found for each of these combinations using a specific yield function and hardening law. As a result, a point cloud is obtained for each functional parameter in the *n*-dimensional space spanned by the process variables. By fitting a suitable equation to this data, an analytical expression depending on the process variables is determined for each functional parameter of the yield function and hardening law. By reinserting these solutions into the original equations, all functional parameters are replaced and a formulation of the THTB yield function and hardening law is obtained. It is obvious, that fitting of the equations must be treated with great precaution to ensure high conformity of the fit with the experimental input. The final THTB material model is obtained by combining the THTB yield function with the hardening law to ensure their compatibility. For example, if the yield parameter in the yield function is defined as one of the functional parameters of the hardening law, it must be ensured that the same polynomial equation is used in both expressions to guarantee consistency of the data. The resulting THTB material model then consists of scalable functions solely dependent on the process variables. The parameters show a quasi-linear correlation with saturation at the borders. Therefore, for the modeling, a quadratic trial function is recommended combining a low number of variables with the necessary accuracy. Based on the interpolation approach, very good agreement with the experimental results can be realized within the technical borders. For the post-forming analysis a forming limit curve is calculated with the MK model that requires a yield function and hardening law as input. The MK model is based on the idea that failure of a specimen under load occurs due to a structural or a geometrical inhomogeneity. In the model, this is idealized by a localized and abrupt decrease in blank thickness, e.g., a groove. The resulting thickness ratio is termed as the inhomogeneity parameter. Considering a plane stress state and that no tangential stresses occur, the load is iteratively increased and the strain state inside and outside of the groove is evaluated. As soon as the ratio of thickness change becomes infinitely large, the failure point of the specimen is reached. By varying the loading condition and tilting the groove, the forming limit is calculated for various strain paths. To ensure the consistency of the THTB model, it must be ensured that the same yield function and hardening law is used as before. The suggested approach provides an easy way to fully describe the material behavior of THTB and allows high flexibility regarding available yield functions and hardening laws.

Since the achievable accuracy is defined by the total number of experiments, the overall experimental effort must still be considered as high. This is especially true, if a large number of process variables should be implemented in the model. Although it seems possible to integrate a high number of variables from a mathematical point of view, fitting of the polynomials can become challenging and must be handled with care. The correlation of the fit function with experimental values should be checked for each expression. In case of uncertainties in the fit, it can be advantageous to selectively increase the experimental input to further specify the respective areas. Furthermore, the model shows high flexibility with regard to implementation in simulation. This can either be realized by simply assigning different material datasets to the locally heat treated blank areas, or by simulating the heating process itself and reversely creating a mechanical property distribution out of the thermal data. Considering a numerical implementation of the THTB model into an existing FE-code, the impact of the introduced variables on the stress update algorithm must be considered. An example of such a modification can be found in Peters *et al.* [17].

### 2.2. Application of the THTB Material Model to AA6014PX 

In the following section, the previous introduced model is applied to an AA6014PX alloy. The maximum temperature and ageing time are considered as process variables being the most important influencing parameters. As experimental input uniaxial tensile tests under various angles to the rolling direction [18], bulge tests [19], biaxial tensile tests [20] and Nakajima tests [21] are used. The heat treatment of all samples was carried out by conduction with a heating time of 3 s. The maximum temperature was varied between 25 and 400 °C and ageing times between 15 min and 6 h. The deviation of the adjusted temperatures caused by the heating equipment is ±5 °C. First, the T4-state is analyzed to identify a yield function and hardening law that describes the experimental data as well as possible. The used mathematical functions are summarized in Appendix A and Appendix B. In a second step, the functional parameters of the yield function and hardening law are expressed depending on the maximum temperature and the ageing time to obtain the THTB material model.

#### 2.2.1. Analysis of the Yield Surface and Flow Curve of the State T4

**Figure 3:**
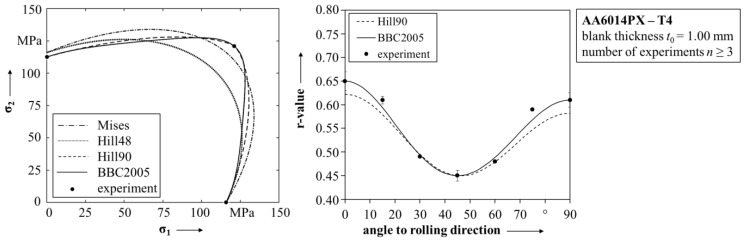
Overview of different yield functions to describe the yield surface of AA6014PX in state T4 and a comparison of the predicted change in r-value for Hill90 and BBC2005.

In Figure 3, the yield functions following Mises [22], Hill48 [12], Hill90 [23] and BBC2005 [13] are shown for the state T4. The yield functions of Mises and Hill48 show high deviations to the experimentally determined yield strengths, especially under biaxial loading. As a consequence, both models are not considered in the following section. In contrast to that, the BBC2005 and Hill90 yield functions are in very good agreement with the experiment. To decide whether the one or the other should be used in the THTB model, the r-value is calculated for load under various angles to the rolling direction and compared with experimental data. The r-value characterizes the anisotropy of the material and thereby influences the material flow during the forming operation. The corresponding results are also shown in Figure 3. Although both models show high conformity with the experimental data, the BBC2005 yield function is slightly closer to the experimental values and is thus used for further analysis. The high correlation to the experiment is a strong indication that the chosen model is an accurate description of the yield surface in the σ_1_-σ_2_ stress space. The numerical solution of the BBC2005 yield function for the state T4 is given in Table C1 in the Appendix.

**Figure 4:**
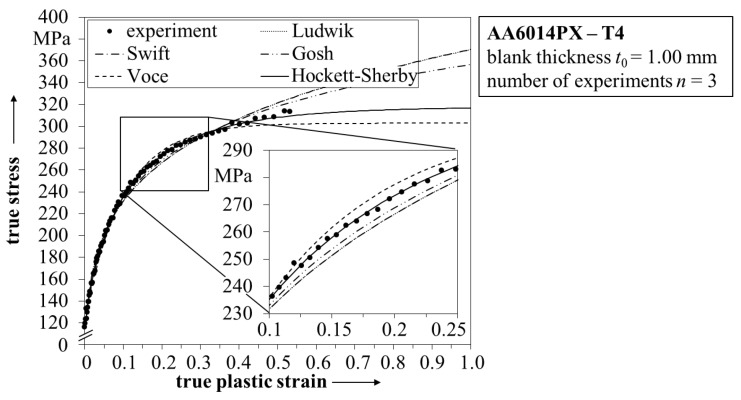
Overview of different hardening laws applied to the flow curve of AA6014PX in state T4.

In Figure 4 the flow curve of the state T4 is shown including fitted hardening laws from Ludwik [24,25], Gosh, Voce [26], Swift [27] and Hockett-Sherby [15]. The experimental data are derived from a bulge test to ensure strain data over the limitations of a conventional uniaxial tensile test. The transformation of the stress-strain data is conducted according to DIN EN ISO 16808 [19]. All hardening laws show high correlation with the experimental data for true strains smaller than 0.1. However, for larger strains deviations of the models become clearly visible.

The inset in Figure 4 shows that the highest correlation with the experiment is obtained by applying the Hockett-Sherby hardening law. In addition, evaluating the extrapolation of the flow curve to true strains of 1 suggests that the model of Gosh overestimates the hardening behavior, while in contrast the model of Voce underestimates the stress increase. The second best correlation to the experimental data is obtained by the hardening law of Swift. This is in accordance to literature, where for a comparable aluminum alloy a combined hardening law of Hockett–Sherby and Swift was used to model the flow curve with high accuracy [28]. At this point, it should also be noticed that there are more advanced approaches available to identify the post-necking hardening behavior [29] based on disentangling the descriptions for pre- and post-necking hardening [30]. However, in this work solely the Hockett–Sherby law is used for the THTB model, due to its very high correlation with the experimental data.

#### 2.2.2. Determination of the THTB Material Model

**Figure 5:**
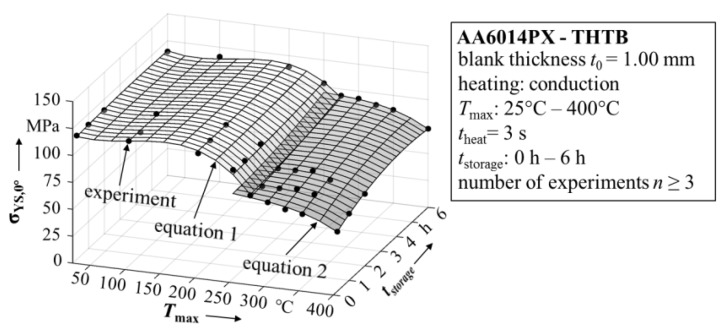
Reference yield strength based on maximum temperature and ageing time.

The BBC2005 yield function is defined by a set of eight non-linear equations. Consequently, eight input parameters are also necessary to identify the yield surface. The functional parameters used in this work and the corresponding methods for their determination are summarized in Table A1 in the appendix. As suggested previously, in principle all these parameters must be expressed as a function of the process variables, namely the maximum temperature and ageing time. However, to minimize the experimental effort one should be aware how the yield surface is influenced by the parameters and the process variables. For example, since the yield function is normalized, its shape only changes when the ratio of stresses or the r-values are varied. In this work, both the stress ratios and r-values remained nearly constant for all tested combinations of maximum temperature and ageing time. This behavior has already been observed in previous investigations on the yield surface of an AA6016 alloy [31]. Therefore, it seems justifiable to use the same normalized yield function for the THTB model. Based on the maximum temperature and the ageing time, the yield surface can thus be written as:

σ_v_(σ*_ij_*,*k_i_*) − *YS*_ref._(*T_max_*,*t*_storage_) = 0 (1)

where σ_v_(σ*_ij_*,*k_i_*) is the normalized yield function and *YS*_ref._ is a polynomial describing the change of the reference yield strength with maximum temperature and ageing time. In other words, to represent the softening correctly, only the reference yield strength must be expressed by a polynomial. This is based on the assumption of isotropic work hardening. To consider a change in the shape of the yield locus as a function of plastic deformation, it is necessary to express all its input parameters by a polynomial equation based on the process variables. In this work the yield strength under load parallel to the rolling direction (σ*_YS_*_,0°_) is used as reference yield strength. The resulting polynomial function is illustrated in Figure 5 together with the experimental data that was used for creating the fit.

**Figure 6:**
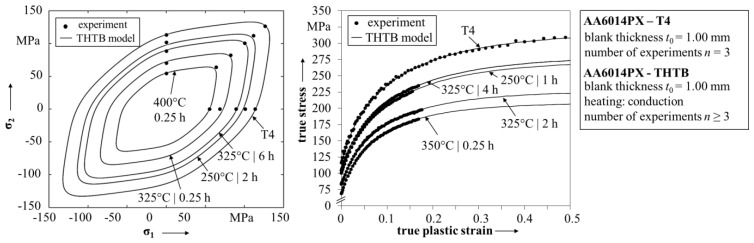
Predicted yield surfaces and flow curves of the THTB material model.

In this work, the highest correlation with the experiment was obtained by using a polynomial order of three for the temperature-related terms and an order of two for the terms describing the change with ageing time. As criterion to evaluate the goodness of the fit, the adjusted *R*^2^-value was used and presumed to be larger than 0.95. To further improve the accuracy of the fit, the dataset was divided at a maximum temperature of 275 °C and two separate fits were created. At a temperature of 275 °C, a change of the microstructural softening effects can be identified. In particular, the combination of the dissolution of GP-Zones in combination with the formation of the β’’-precipitations leads to a discontinuity in the mechanical property change [4]. With both fit functions the dataset could be described sufficiently accurate within the range of the experimental data points. Reinserting the polynomial fit for the reference yield strength into Equation (1), leads to a description for the THTB yield surfaces. In Figure 6, the results of the THTB model are plotted together with experimental values for various combinations of maximum temperature and ageing time. The model shows high conformity with the experimental data and describes the physical behavior of THTB in agreement to microstructural processes as given in literature. For example, the yield surface is shrinking with increasing maximum temperature due to softening of the material (*cf.* yield surface for state T4 and *T_max_* = 400 °C, *t*_storage_ = 0.25 h), while for a constant maximum temperature prolonged ageing leads to an expansion of the yield surface (*cf.* *T_max_* = 325 °C, *t*_storage_ = 0.25 and 6 h).

The Hockett-Sherby law is defined as a function of true strain as:

σ(ε*_w_*) = σ_sat._ − (σ_sat._ − σ*_YS_*_,0°_)∙exp(−*C*∙ε*_w_^D^*). (2)

Hereby, the flow curve is defined by a saturation strength σ_sat._, the yield strength σ*_YS_*_,0°_ and two additional functional parameters C and D, which are mainly used to describe the hardening behavior. Again, all parameters must be expressed as a function of maximum temperature and ageing time. While it was possible to model the change in yield surface by only one parameter, the hardening law for THTB needs a more careful treatment. Firstly, to ensure consistency of the data, the yield strength σ*_YS_*_,0°_ should be described by the same expression as used for the yield surface, *cf.* Figure 5. Furthermore, Vogt indicated that the parameters C and D are strongly interconnected with each other and thus suggested to set parameter D to a constant value defined by the flow curve of the state T4. This approach is also used in this work. Consequently, only the saturation strength and parameter C remain to be described by a suitable equation. As mentioned previously, the best fit to the experimental data is evaluated using the adjusted *R*^2^-value, which is presumed to be larger than 0.95. As for the yield strength, to improve the accuracy the dataset was again divided at 275 °C and two separate fit functions were created. Reinserting the solutions into Equation (2) leads to a scalable flow curve function for THTB which depends on the maximum temperature and ageing time. The prediction of the model and experimentally obtained values are shown in Figure 6. The detailed equations and fitting parameters are listed in Table C2 in the appendix. The model describes the flow curves for different combinations of maximum temperature and ageing time in high agreement with the experiment. Even small differences between the flow curves, like for *T_max_* = 325 °C, *t*_storage_ = 4 h and *T_max_* = 250 °C, *t*_storage_ = 1 h, are correctly reproduced.

**Figure 7:**
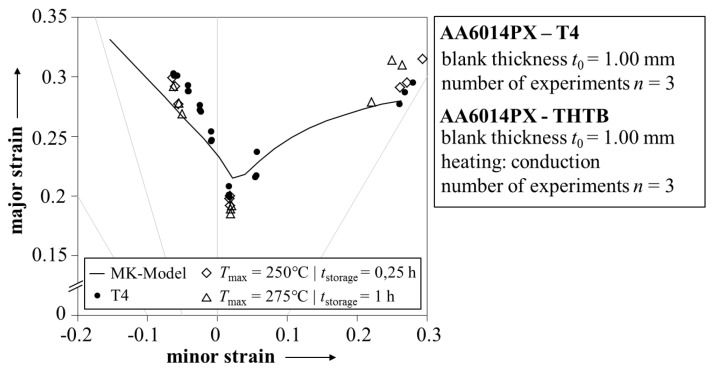
Experimental and calculated forming limit curve of AA6014PX.

The determined THTB yield function and hardening law fully describe the material behavior during plastic deformation. Besides, a forming limit curve is needed for the failure analysis. The MK model was used for the calculation with BBC2005 and Hockett-Sherby as input for the yield function and hardening law, respectively. The imperfection parameter was set to 0.997. The resulting forming limit curve for the state T4 is shown in Figure 7 with experimental values obtained in Nakajima tests. The calculated forming limit curve is generally in good agreement to the experiment, but shows some slight deviations in the range of negative minor strains. However, considering the spread per geometry, this difference can most likely be explained as a matter of statistics.

In addition, the experimentally determined forming limits of two THTB are also shown in Figure 7. No evident difference in forming limits of the THTB is observed in comparison to the state T4. At first glance, this is unexpected since a reduction in uniform elongation is reported for maximum temperatures in the range from 225 to 300 °C [4]. To address this behavior in modeling, it is suggested that the forming limit curve is shifted to lower major strains proportionally to the reduction [32]. However, on the basis of the available data in this work, it is not possible to derive a constant of proportionality. This leads to the assumption that the shift is not distinctive for the investigated alloy, although it has been clearly observed for other 6xxx-aluminum alloys in the past [33]. For the validation of the THTB model, it is thus concluded that the result from the MK model (T4) is also applicable for the various THTB conditions.

## 3. Process Design of Aluminum Tailor Heat Treated Blanks

**Figure 8:**
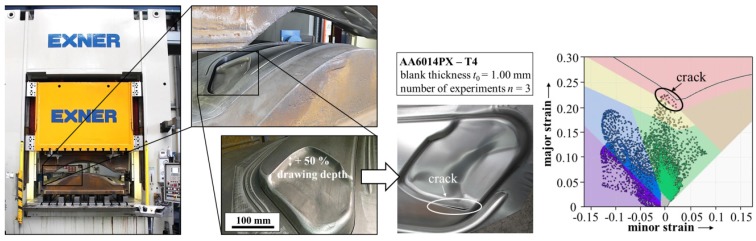
Press including the forming tool for production of the tailgate with 50% increased drawing depth in the area of the light fixture.

The above-described approach was used for the process design of a THTB application. In order to validate the technology, a complex tailgate geometry was chosen. Figure 8 shows the forming press, including the forming tool, for the production of the tailgate. For aluminum alloys, this tool represents a state-of-the-art geometry as nowadays used for exposed applications in passenger cars. In addition, the drawing depth in the area of the light fixture is increased by 50% to demonstrate the capability of the THTB technology. With a conventional aluminum sheet in T4 state, the tailgate cannot be formed without showing a crack at the edge of the radius region. This is illustrated in Figure 8, together with the corresponding formability results from numerical simulation realized with AutoForm R5.2 (AutoForm Engineering GmbH, Wilen, Switzerland). Thereby, the above-described material model was implemented. The tests were performed with a blank holder force of 200 kN and without the application of a forming lubricant. For the investigation, an aluminum alloy, AA6014PX, with a sheet thickness of 1 mm was applied. The experimental setup is used as input for the forming simulation and transferred as close to reality as possible, e.g., tool geometries, blank outline, blankholder force, *etc.* Besides, the restraining forces are calculated by an adaptive model that is based on the drawbead geometry.

In order to improve the formability of the aluminum alloy and produce the part without failure, the local heat treatment approach should be used. Most challenging within the process design of the THTB technology is the definition of suitable heat treatment layout. As previously indicated by the comprehensive material characterization, the short-term heat treatment does not directly lead to an enhancement of the formability. On the contrary, if the crack critical area is directly heated, the part would even be more susceptible to failure [4]. Therefore, an indirect approach is applied. Based on the local heat treatment and the resulting property distribution, the material flow can be influenced by the interaction of soft and hard areas.

During the design procedure of a suitable heat treatment, the crack critical areas are first identified by the experimental and numerical results. In particular, the sharp edges of the forming tool lead to a localization of the plastic strains and finally to an early failure. This is also indicated by the FE-simulation and the used failure criteria.

**Figure 9:**
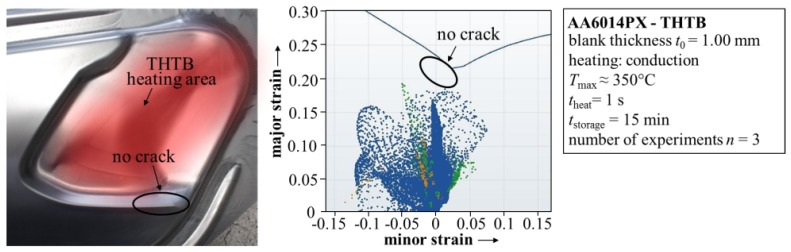
Overview of the tailgate without failure produced by forming of a THTB together with failure analysis results from simulation.

In order to improve the material flow to the sharp edge and thereby reduce the critical strains, a relief zone has to be defined. The relief zone should, on one hand, be in the immediate vicinity of the crack critical area. However, on the other hand, the relief zone itself should not be critical to failure. The short-term heat treatment of the relief zone leads to a dissolution of the co-clusters in the microstructure, resulting in lowered yield strength compared to the state T4. Consequently, these areas begin to plastify earlier during the forming operation and the material flow to the crack critical area is improved. In Figure 9, the heat treated area for the tailgate zone is presented in red. Moreover, it can be seen that the part can now be produced without failure by the application of the THTB technology.

During process design, the mechanical property distribution of THTB should be optimized with the objective to reduce the occurring critical strains during the forming operation. For example, in simulation this can be realized by evaluating the failure value for different heat treatment layouts until the minimum is reached. However, one should be aware that the best forming result can only be achieved by a combination of optimum blank outline and heat treatment layout [8]. This might represent a constraint of the optimization in case it is not possible to change the blank outline, e.g., due to following process steps or production costs.

**Figure 10:**
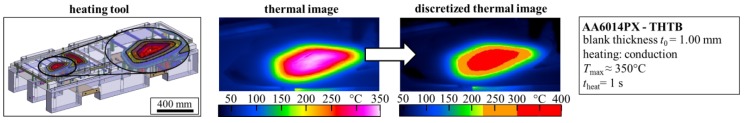
Heating tool and experimentally determined temperature distribution (non-discretized and discretized) of the blank at the moment that the maximum temperature is reached during the heating process.

For the heat treatment, different technologies based on heat induction, heat conduction and laser radiation can be used. A flexible robot controlled laser system is advantageous for the design of various heat treatment layouts, because the laser path can be easily changed. However, the heating time and the necessary energy are high. Moreover, the homogenous and residual stress free heat treatment of large areas is quite challenging; consequently, a laser system is recommended, in particular, for the heat treatment of small areas within a prototype phase. For the described part, a relatively large area with a small heat affected zone should be heat treated. Therefore, a conductive heating approach was chosen. In Figure 10, the developed tool is presented.

The tool was implemented into a second hydraulic press directly before the actual forming press. The tool was designed with two different heating areas, which were of particular interest for the study of the heat affected zones and the material characterization. However, for the production of the THTB parts, only the left heating zone was used. The comprehensive material characterization indicated that the maximum temperature is the most important parameter for influencing the mechanical properties. Keeping in mind the necessary localization of the heat treatment, the heating time should be as short as possible. In order to realize fast heating, a cartridge heater was integrated into the tool. Thereby, a heating time of approximately one second can be achieved to reach the maximum temperature of 350 °C. Due to the shortness of the heat treatment, it is energy efficient.

The forming step of the THTB was performed after a storage time of 15 min at room temperature. In contrast to conventional temperature assisted forming processes, the aluminum alloy cools down completely before the forming operation. Therefore, in addition the conventional cold forming tool can be used without any changes. Figure 10 shows the heating tool together with the resulting temperature distribution of the blank at the moment the maximum temperature is reached. The thermal image was obtained experimentally using an infrared camera with an accuracy of ±5 °C. Furthermore, for a successful process design, correct positioning of the blanks during the heat treatment was ensured with positioning bolts.

As a next step, the thermal image is discretized by specific temperature levels for identifying the areas of the blank in which the same material properties apply. These areas are transferred to the FE-simulation and the above-presented model is used to specify the respective material properties. Based on this local softening, the maximum effective strains at the sharp edge are reduced and the tailgate produced from the THTB can be classified as “non-critical” (*cf*. Figure 9). In comparison to forming of the state T4 blank, the maximum failure value in the crack critical region is reduced from around 0.93 to 0.74.

**Figure 11:**
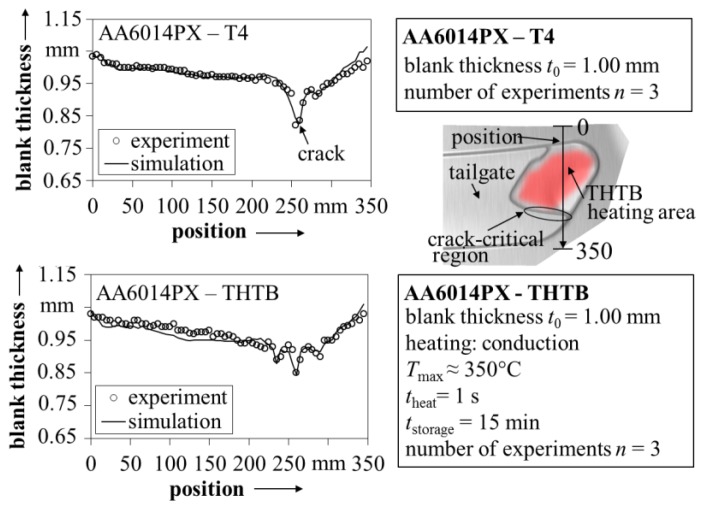
Comparison of blank thickness from simulation with experimental data for the state T4 and THTB.

In addition, within the validation of the scientific procedure and the developed material model, a comparison of measured and calculated thicknesses is shown in Figure 11 for tailgates produced from the state T4 and THTB. As indicated in the overview geometry, the data refers to positions where high forming forces occur as well as to heating areas of the THTB. The predicted blank thickness is in very high agreement with the experimental data from the measurements using a gauge with tactile probes. Although not shown here, the high correlation was also confirmed for various other positions on the blank. This demonstrates the capability of the suggested material model to describe the behavior of THTB during complex forming operations.

**Figure 12:**
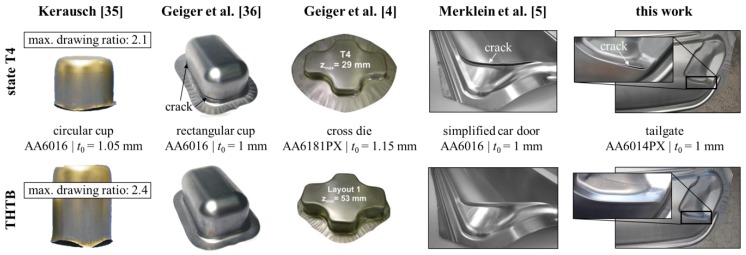
Overview of different geometries that have been used to demonstrate the enhancement of the forming limits by the THTB technology.

With regard to lightweight construction, the presented experiments show the potential of the THTB technology to significantly improve the forming limits. This is of particular interest to the automotive industry, since 6xxx-series alloys are widely used in exposed applications and thus in direct competition with the superior formable mild or high strength steels [34]. The enhancement of the forming limit has been demonstrated before for different geometries with varying complexity, e.g., circular cup [35], rectangular cup [36], cross-die [4] or a simplified car door [5]. This is illustrated in Figure 12 together with the tailgate from this work. For example, the maximum drawing depth of the cross-die was increased by around 86% when using the THTB technology in comparison to forming of the state T4. However, the application of THTB was so far mostly limited to parts with a high deep-drawing share [37]. The tailgate used in this work represents the first application of THTB in combination with a complex, outer panel geometry involving various strain states and non-linear strain paths. In addition, the above-presented material model enables a reliable numerical prediction of the forming operation. This opens the possibility to evaluate the use of THTB during process planning without expensive trial-and-error procedures. In this regard, it should be kept in mind that the validity of the THTB model was so far only shown for the tailgate and the possibility of its transformation to other complex geometries must still be investigated.

## 4. Summary and Outlook

A holistic process design approach is introduced for the application of the THTB technology to complex forming parts. The model is exemplarily applied on the experimental data of an AA6014PX alloy based on the maximum temperature and ageing time. The capability of the model to describe complex forming operations is demonstrated on a tailgate by comparison of the thickness data from the simulation with the experimental results. Thereby, it was demonstrated that the THTB technology can be used to significantly improve the formability of aluminum alloys. Moreover, there is an excellent agreement between the numerical and experimental results. In the future, the performance of the developed approach will be applied to further precipitation hardenable aluminum alloys and stamping parts.

## Appendix A: Yield Functions

Mises [19]:

$${\left(\sigma_{1}-\sigma_{2}\right)}^{2}+\sigma_{1}^{2}+\sigma_{2}^{2}=2\sigma_{0}^{2}$$

Hill48 [12]:

$$\sigma_{1}-\frac{2r_{0}}{1+r_{0}}\sigma_{1}\sigma_{2}+\frac{r_{0}\left(1+r_{90}\right)}{r_{90}\left(1+r_{0}\right)}\sigma_{2}^{2}=\sigma_{0}^{2}$$

Hill90 [20]:

$$|\sigma_{1}+\sigma_{2}{|}^{m}+\left(\frac{\sigma_{b}^{m}}{\tau^{m}}\right){\left|\sigma_{1}-\sigma_{2}\right|}^{m}+{\left|\sigma_{1}^{2}+\sigma_{2}^{2}\right|}^{\left(m/2\right)-1}\left(-2a{\left(\sigma_{1}-\sigma_{2}\right)}^{2}+b{\left(\sigma_{1}-\sigma_{2}\right)}^{2}\right)={\left(2\sigma_{b}\right)}^{m}$$

where:

$$\left(\frac{\sigma_{b}^{m}}{\tau^{m}}\right)=1+2r_{45}\;a=\frac{1}{4}\left[{\left(\frac{2\sigma_{b}}{\sigma_{90}}\right)}^{m}-{\left(\frac{2\sigma_{b}}{\sigma_{0}}\right)}^{m}\right]\;b=\frac{1}{2}\left[{\left(\frac{2\sigma_{b}}{\sigma_{0}}\right)}^{m}+{\left(\frac{2\sigma_{b}}{\sigma_{90}}\right)}^{m}\right]-{\left(\frac{2\sigma_{b}}{\sigma_{45}}\right)}^{m}$$

BBC2005 [13]:

$$\sigma_{0}={\left[a{\left(\Lambda +\Gamma \right)}^{2k}+a{\left(\Lambda -\Gamma \right)}^{2k}+b{\left(\Lambda +\Psi \right)}^{2k}+b{\left(\Lambda -\Psi \right)}^{2k}\right]}^{1/2k}$$

where:

$$\Gamma =L\sigma_{11}+M\sigma_{22}$$

$$\Psi =\sqrt{{\left(N\sigma_{11}-P\sigma_{22}\right)}^{2}+\sigma_{12}\sigma_{21}}$$

$$\Lambda =\sqrt{{\left(Q\sigma_{11}-R\sigma_{22}\right)}^{2}+\sigma_{12}\sigma_{21}}$$

**Table A1:** Overview of the parameters used for the calibration of the BBC2005 yield surface.

Variable	T4 value	Description	Method
σ*_YS_*_,0°_	115 ± 1 MPa	yield strength under uniaxial loading for 0°/45°/90° to the rolling direction	uniaxial tensile test [18]
σ*_YS_*_,45°_	116 ± 1 MPa		
σ*_YS_*_,90°_	112 ± 2 MPa		
σ*_YS_*_,*b*_	121 ± 3 MPa	yield strength under biaxial loading (σ_1_ = σ_2_, σ_3_ = 0)	biaxial tensile test [19]
r_0°_	0.65 ± 0.019	anisotropy value under uniaxial loading for 0°/45°/90° to the rolling direction	uniaxial tensile test [18]
r_45°_	0.45 ± 0.015		
r_90°_	0.61 ± 0.015		
r*_b_*	0.92 ± 0.025	anisotropy value under biaxial loading (σ_1_ = σ_2_, σ_3_ = 0)	layer compression test [38]

## Appendix B: Flow Curves

Ludwik [21,22]:

$$\sigma_{w}=\sigma_{0}+k\varepsilon_{w}^{n}$$

Swift [24]:

$$\sigma_{w}=k{\left(\varepsilon_{w}+\varepsilon_{0}\right)}^{n}$$

Ghosh:

$$\sigma_{w}=k{\left(\varepsilon_{w}+\varepsilon_{0}\right)}^{n}-c$$

Voce [23]:

$$\sigma_{w}=\sigma_{\infty }-\left(\sigma_{\infty }-\sigma_{0}\right)\text{exp}\left(-k\varepsilon_{w}\right)$$

Hockett–Sherby [15]:

$$\sigma_{w}=\sigma_{\infty }-\left(\sigma_{\infty }-\sigma_{0}\right)\text{exp}\left(-k\varepsilon_{w}^{n}\right)$$

## Appendix C

**Table C1:** Solution of the BBC2005 yield criterion for the state T4. Variables are named according to [13].

Variable	a	M	N	P	Q	R	S	T
T4-solution	1.3758	0.2351	0.3990	0.4449	0.5115	0.5018	0.5395	0.5591

The following two polynomial equations were used in this work:

Polynomial_32 = p00 + p10 *T_max_* + p01 *t*_storage_ + p02 *T_max_*^2^ + p11 *T_max_·t*_storage_ + p02 *t*_storage_^2^ + p30 *T_max_*^3^ + p21 *T_max_*^2^*·t*_storage_ + p12 *T_max_·t*_storage_^2^

Polynomial_33 = p00 + p10 *T_max_* + p01 *t*_storage_ +p02 *T_max_*^2^ + p11 *T_max_·t*_storage_ + p02 *t*_storage_^2^ + p30 *T_max_*^3^+ p21 *T_max_*^2^*·t*_storage_ + p12 *T_max_·t*_storage_^2^ + p03 *t*_storage_^3^

**Table C2:** Overview of the parameters obtained by polynomial fitting.

Parameter	σ*_YS_*_,0°_		σ_sat._		*C*	
	Fit 1	Fit 2	Fit 1	Fit 2	Fit 1	Fit 2
p00	117.3	215.2	314.7	335.0	6.067	−27.36
p10	−0.2086	−1.365	−0.2422	0.1494	−0.01509	0.2996
p01	1.949	−17.73	3.783	−136.8	0.1234	1.666
p20	0002894	0.00475	0.005042	−0.003417	3.536 × 10^−9^	−8.428 × 10^−4^
p11	−0.02748	0.07863	−0.02099	0.7111	−8.024 × 10^−4^	−0.01783
p02	−0.1993	2.775	–0.4793	6.53	−0.018	0.4022
p30	−9.567 × 10^−6^	−5.877 × 10^−6^	−1.906 × 10^−5^	5.53 × 10^−6^	2.386 × 10^−7^	7.725 × 10^−7^
p21	8.671 × 10^−5^	−2.659 × 10^−5^	5.829 × 10^−5^	8.257 × 10^−4^	−2.521 × 10^−6^	3.06 × 10^−5^
p12	0.001369	−0.009505	0.001415	−0.0202	1.891 × 10^−4^	−3.901 × 10^−4^
p03	-	-	-	-	-	−0.02772
